# PARP1 Promotes Heart Regeneration and Cardiomyocyte Proliferation

**DOI:** 10.7150/ijbs.85526

**Published:** 2024-02-11

**Authors:** Jiangcheng Shu, Shu Yan, Chenhui Ju, Long Chen, Minglu Liang, Cheng Wang, Kai Huang

**Affiliations:** 1 Department of Cardiology, Union Hospital, Tongji Medical College, Huazhong University of Science and Technology, Wuhan, Hubei, China.; 2 Clinic Center of Human Genomic Research, Union Hospital, Tongji Medical College, Huazhong University of Science and Technology, Wuhan, Hubei, China.; 3 Department of Rheumatology, Union Hospital, Tongji Medical College, Huazhong University of Science and Technology, Wuhan, Hubei, China.

**Keywords:** cardiomyocyte proliferation, heart regeneration, cell cycle, cell signaling, PARP1

## Abstract

Myocardial infarction causes cardiomyocyte loss, and depleted cardiomyocyte proliferative capacity after birth impinges the heart repair process, eventually leading to heart failure. This study aims to investigate the role of Poly(ADP-Ribose) Polymerase 1 (PARP1) in the regulation of cardiomyocyte proliferation and heart regeneration. Our findings demonstrated that PARP1 knockout impaired cardiomyocyte proliferation, cardiac function, and scar formation, while PARP1 overexpression improved heart regeneration in apical resection-operated mice. Mechanistically, we found that PARP1 interacts with and poly(ADP-ribosyl)ates Heat Shock Protein 90 Alpha Family Class B Member 1 (HSP90AB1) and increases binding between HSP90AB1 and Cell Division Cycle 37 (CDC37) and cell cycle kinase activity, thus activating cardiomyocyte cell cycle. Our results reveal that PARP1 promotes heart regeneration and cardiomyocyte proliferation via poly(ADP-ribosyl)ation of HSP90AB1 activating the cardiomyocyte cell cycle, suggesting that PARP1 may be a potential therapeutic target in treating cardiac injury.

## Introduction

Myocardial infarction (MI) and subsequent heart failure (HF) continue to be significant contributors to morbidity and mortality on a global scale^1^. Since the mammalian heart has low endogenous regenerative capacity, the infarcted heart undergoes postinfarct remodeling and eventually degenerates, leading to heart failure. However, neonatal mice are able to repair heart injury and regenerate damaged myocardium, while their capacity for regeneration diminishes 7 days after birth^2^. Interestingly, recent work has begun to reveal that adult mammalian hearts possess proliferative and regenerative properties under certain circumstances, raising hope for therapeutic approaches to repair damaged hearts by promoting endogenous cardiac regeneration.

Poly(ADP-ribose) polymerase 1 (PARP1) is a prominent member of the PARP family. Its primary function is to transfer ADP-ribose polymer chains from NAD^+^ to acceptor proteins or PARP1 protein itself, called posttranslational poly(ADP-ribosyl)ation modification^3^. PARP1 participates in a multitude of cellular processes, such as transcriptional activation, cell death, chromatin remodeling, and energy metabolism^4^. Increasing evidence has indicated the involvement of PARP1 in a range of cardiovascular diseases, such as cardiac hypertrophy, arrhythmia, atherosclerosis, hypertension, and vascular calcification^5^-^9^. Recently, research showed that PARP1 contributes to stem cell maintenance and differentiation by modulating embryonic stem cell gene activity^10^. However, there is a lack of comprehensive understanding regarding the function of PARP1 in cardiomyocyte proliferation and heart regeneration.

This study aims to examine the involvement of PARP1 in the process of cardiomyocyte proliferation and its potential implications for heart regeneration. We hypothesize that PARP1 may act as a key effector in neonatal heart development, and PARP1 activation promotes heart regeneration and cardiomyocyte proliferation mainly mediated by poly(ADP-ribosyl)ation.

## Methods

### Mice

The animal procedures adhered to the guidelines set forth by the National Institutes of Health guide for the care and use of Laboratory animals and received approval from the Ethics Committee of Union Hospital, Huazhong University of Science and Technology (Wuhan, China). The study utilized male C57BL/6J mice aged 8-10 weeks, which were obtained from Vital River Laboratory Animal Technology Co. Ltd. (Beijing, China). PARP1 knockout mice and their WT littermates, Myh6-Cre were obtained from the Jackson Laboratory (Bar Harbor, Maine, USA). IACUC number was 3022 for animal experiments.

The generation of PARP1 conditional knockin mice was carried out by Cyagen Biosciences Inc. (Guangzhou, China). A schematic representation of the conditional knockin of PARP1 was shown in **Figure S1**. In detail, we constructed a donor vector containing “CAG promoter-loxP-PGK-Neo-6*SV40 pA-loxP-Kozak-Human PARP1 CDS-rBG pA” cassette, which is cloned into intron 1 of Rosa26 locus. A co-injection approach was employed by introducing the gRNA targeting the mouse ROSA26 gene, the donor vector, and Cas9 mRNA into fertilized mouse eggs. PCR and sequence analysis were performed for verification purposes of the F0 founder mice, which were crossbred with WT mice to examine the generation of F1 mice. Following numerous generations of reproductive crosses, we mated ROSA26-LSL-PARP1 with Myh6-Cre mice to generate cardiomyocyte-specific PARP1 knockin (PARP1^CKI^) mice.

### Apical resection (AR)

P1 and P7 neonatal mice were subjected to AR operation as described^11^-^13^. Neonatal mice were randomly chosen and then anesthetized on ice bed cooling for approximately 2-3 minutes. After anesthetization, mice underwent a transverse skin incision across the lower half of the left chest. The intercostal muscles were separated at the third or fourth intercostal space to expose the inner chest cavity. The mouse heart was exteriorized by gently pressing the mouse abdomen. Once exteriorized, 10%-15% of heart apex tissue was resected until the left ventricular chamber was exposed by using iridectomy scissors. After resection, the skin incision was sutured, and mice were immediately placed on a heating pad for resuscitation. The overall procedure should be finished within 10 minutes. Sham mice were operated with the same procedure except for apical resection. 7 and 21 days postresection (dpr), mice were euthanized by intraperitoneally injecting sodium pentobarbital (200 mg/kg), and hearts were obtained or fixed in 4% paraformaldehyde for subsequent experiments.

### Myocardial infarction (MI)

Adult WT mice were randomly assigned to several groups and subjected to MI as previously described^11^, ^14^, ^15^. All adult mice were subjected to anesthesia using sodium pentobarbital (50 mg/kg) and maintained under ventilation throughout the entire procedure. The chest was exposed, and a 7-0 nonabsorbable silk suture was used to ligate the left anterior descending (LAD) coronary artery. The sham mice were subjected to the same procedure, except for LAD ligation. Once the LAD is ligated, AAV9-PARP1 or AAV9-Ctrl was intramyocardially injected into the myocardium bordering the infarct zone at three locations (left, right, and top) by using a micro syringe with a 33 G needle (3 μl for each point). 7 days and 28 days post MI (dpi), Pentobarbital sodium (200 mg/kg) was used for euthanasia via intraperitoneal injection, and hearts were obtained for subsequent experiments.

### Echocardiography

Cardiac function was evaluated before, 3 days and 28 days after MI by echocardiography using the Vevo 770 VisualSonic echocardiographic imaging system (VisualSonic, Canada). Mice were firstly placed in a supine position after anesthetization, and cardiac function was measured using two-dimensional (2D)-targeted M-mode and B-mode images, obtained by applying an ultrasonic probe to the left midventricular region. Ejection fraction (EF) and fractional shortening (FS) were automatically estimated by VisualSonic software (VisualSonic, Canada).

### Histology

Heart tissues were collected and fixed in 4% paraformaldehyde overnight, embedded in paraffin wax, and sectioned for indicated experiments. Masson trichrome staining was conducted using the Trichrome Stains (Masson) kit (HT15, Sigma-Aldrich, USA) to evaluate collagen according to the manufacturer's protocol. Scar size in the MI model and AR model were calculated by the scar area (collagen stained blue) versus the total left ventricular area by ImageJ (NIH, Bethesda, USA)^11^, ^14^. WGA staining was performed using WGA staining kit (W11261, Thermo Fisher, USA) to measure the cross-sectional area of cardiomyocytes following the manufacturer's instructions. Evans blue-TTC staining (IE0280, T8170, Solarbio, China) was also performed according to the manufacturer's instructions, and AAR representing area at risk (red area), IS representing infarct size (white area) was measured by ImageJ. Cardiomyocyte cross-sectional area was quantified by ImageJ as previously described^16^, ^17^. Cross-sectioned cardiomyocytes were selected and outlined by using the “freehand selection tool” of ImageJ, and cross-sectional area was calculated by checking “Area” and “Bounding rectangle” options and “Measure” function.

### Primary neonatal rat cardiomyocyte isolation, culture and transfection

Neonatal Sprague‒Dawley (SD) rats were utilized to isolate neonatal rat cardiomyocytes as previously described^15^, ^18^, ^19^. Neonatal rats were euthanized by rapid decapitation, and their hearts were immediately collected and minced. 0.1% type II collagenase (#LS004174, Worthington Biochemical, USA) was used for heart tissue digestion in a glass beaker with gentle stirring at 37 °C for 15 min repeated 3 times. After collection and centrifugation, pellets were separated by a Percoll gradient for isolation of different cell types in the resuspension (3000 rpm, 30 min). The lower band of NRCMs was collected, centrifuged, and resuspended. NRCMs were cultured in wells coated with 0.1% gelatin (G1890, Sigma, USA) for subsequent experiments. For adenovirus treatment, Ad-PARP1 or Ad-Ctrl was used to infect cardiomyocytes for 48 h. Over 95% of cardiomyocytes were transduced, and the cardiomyocytes were harvested at the indicated times as the experiments needed. siRNAs were used to interfere with the expression of PARP1 and HSP90AB1. siRNAs were transfected into cardiomyocytes using bPEI (Sigma, USA) following the manufacturer's instructions. The si-PARP1 target sequence was as follows: sense 5'-GGAUGAUCUUCGACGUGGA-3'; The si-HSP90AB1 target sequence was as follows: sense 5'-CCUCAUAAAUAACUUGGGAACCAUU-3'.

### Western blot

Protein lysates were obtained from heart tissue or NRCMs using RIPA buffer (G2033, Servicebio, China) with PMSF (ST506, Beyotime, China) and phosphatase inhibitor. The concentration of protein was evaluated by BCA protein assay and samples were heated with loading buffer at 99 °C for 10 min for subsequent western blot. The primary antibodies were listed as follows: PARP1 (#9532S), AKT (#4691), phospho-AKT (#4060), and phospho-ERK1/2 (#9101) from Cell Signal Technology (USA), CDC37 (ab109419) and HSP90AB1 (ab203085) from Abcam (USA), PARP2 (55149-1-AP), CDK4 (11026-1-AP), ERK1/2 (16443-1-AP) and GAPDH (60004-1-AP) from Proteintech (China), PAR (4335-MC-100) from R&D (USA). Samples were incubated with secondary antibodies the following day for 2 h. Membranes were imaged by ChemiScope 6000 Series (Clinx Science Instruments Co., Ltd., Shanghai, China) and estimated by ImageJ software (NIH, Bethesda, USA).

### Coimmunoprecipitation (Co-IP)

Heart tissues or cardiomyocytes after treatment were lysed and incubated with the antibody of interest or IgG (#70024s, CST, USA) overnight at 4 °C. Samples were incubated with Protein A/G Magnetic Beads (HY-K0202, MCE, China) for 2-4 h at 4 °C. The lysates were removed, and the beads were washed and heated with loading buffer at 99 °C for 10 min for subsequent western blot.

### Immunostaining

Immunostaining assay was performed as described^20^, ^21^. Heart sections were dehydrated with a dimethylbenzene series and ethanol and subjected to heat-mediated antigen retrieval using EDTA solution. NRCMs were fixed in 4% formaldehyde, and heart sections or NRCMs were then permeabilized with Triton-X (0.1%). Samples were blocked with 5% goat serum (Servicebio Cat: WGAR1009) for 1 h, and incubated with antibodies overnight at 4 °C. EdU assay (C10310-1, Ribobio, China) was applied following the manufacturer's instructions. Antibodies used were: anti-cardiac troponin T (ab8295), anti-phosphohistone H3 (S10) (ab5176), anti-sarcomeric alpha actinin (ab9465), Aurora B (ab2254), BrdU (ab8152), Ki67 (ab16667) from Abcam (USA). Samples were stained with secondary antibodies the next day for 2 h. DAPI (ANT165, AntGene, China) was used for nuclear visualization. A confocal laser scanning microscope (Nikon, Tokyo, Japan) was utilized for sample scanning, and the measurement of stained cell density was conducted by ImageJ software (NIH, Bethesda, USA).

### CDK4 kinase activity assay

CDK4 kinase activity was measured using Cell CDK4/Cyclin D Colorimetry Assay Kit (GENMED Scientifics Inc., USA). Briefly, NRCMs infected with Ad-Ctrl or Ad-PARP1 were washed, and proteins were lysed and mixed with reagents. Optical density values were estimated 5 times with 1 min intervals and recorded by an absorbance microplate reader (Bio-Rad, USA).

### BiFC and FRET

BiFC and FRET assays were performed following the guidance of Shyu, Y.J., et al^22^. HEK293T cells were cultured to 50% confluence for subsequent transfection. For BiFC assay, HEK293T cells were transfected with PARP1-VC155 or HSP90-VN173 or empty vector. For FRET assay, HEK293T cells were transfected with PARP1-CFP, HSP90-YFP, or empty vector. 24-48 h after transfection, BiFC and FRET complex fluorescence was visualized by a confocal laser scanning microscope.

### Statistics

The experiments were conducted a minimum of three times, and data are expressed as the means ± SEM. Before applying parametric tests, Shapiro-Wilk test was utilized to examine the normality of the data distribution. Nonparametric tests were employed to examine statistical differences in non-normal distribution data. The significance of comparisons between two groups was evaluated by either Student's t-test or the nonparametric Mann-Whitney test. In terms of comparing different groups, ANOVA followed by the Bonferroni test was employed for parametric data, and the Kruskal-Wallis test and Dunn's post hoc test were utilized for nonparametric data. An F test was employed for comparing variances for t-tests between two groups, while the Brown-Forsythe test was utilized for comparing variances for ANOVA among many groups.

## Results

### PARP1 expression is restricted in the developing heart

**Figure 1:**
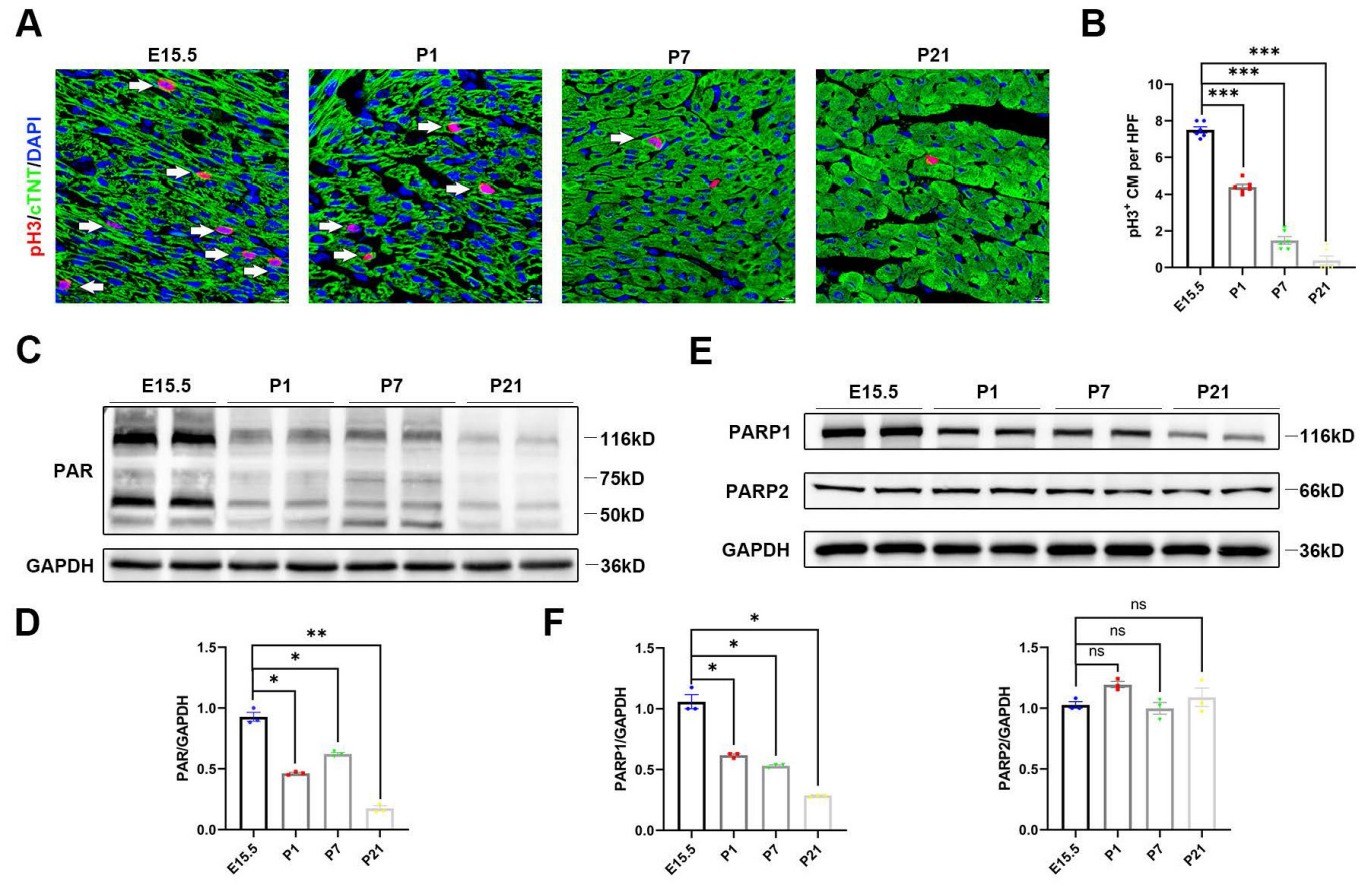
** PARP1 expression is restricted in the developing heart. (A)** Representative immunostaining images of pH3^+^ (red) cardiomyocyte number in C57BL/6J WT mice heart at E15.5, P1, P7, and P21. Scale bars, 10μm. **(B)** Statistical analysis of pH3^+^ cardiomyocytes in C57BL/6J WT mice with heart development (n=6 per group). ***P < 0.001 by one-way ANOVA with Bonferroni's multiple comparisons test. **(C)** Representative western blot images of PAR and GAPDH protein expression from E15, P1, P7, and P21 C57BL/6J WT mice heart. **(D)** Statistical analysis of PAR protein expression with heart development (n=3). *P < 0.05, **P < 0.01 by one-way ANOVA with Bonferroni's multiple comparisons test. **(E)** Representative western blot images of PARP1, PARP2, and GAPDH protein expression from E15.5, P1, P7, and P21 mice hearts. **(F)** Statistical analysis of PARP1 and PARP2 protein expression with heart development (n=3). *P < 0.05 by one-way ANOVA with Bonferroni's multiple comparisons test.

In line with a previous report, cardiomyocyte proliferation by immunofluorescence revealed high levels of myocardial phosphohistone H3 (pH3), a mitosis marker of cell division, on embryonic Day 15.5 (E15.5), which were strongly reduced by P1 and P7 and remained extremely low until postnatal Day 21 (P21) (**Figure 1A** and** 1B**). To explore whether PARP activity was involved in neonatal heart regeneration, we first examined PARP activity at each developmental stage. We found that poly(ADP)ribosylation, which represented PARP activity, decreased in the postnatal hearts by western blot (**Figure 1C** and** 1D**). PARP1 and PARP2 are major catalytically active enzymes that covalently modify poly(ADP)ribosylation of target proteins. We noted that protein expression of PARP1 was gradually reduced in postnatal cardiac tissue, while PARP2 expression was unchanged (**Figure 1E** and** 1F**). We further examined the relationship of PARP1 and PAR expression with cardiomyocyte proliferation by immunostaining. The cardiomyocyte expression of PARP1 and PAR on P21 was significantly reduced compared to that on E15.5 (**Figure S2A** and** 2B**). These observations demonstrated that a reduction of PARP1 paralleled the decline in cardiomyocyte proliferative capacity during neonatal heart development, suggesting that PARP1 may be involved in heart regeneration.

### Knockout of PARP1 inhibits cardiac regeneration in neonatal mice

**Figure 2:**
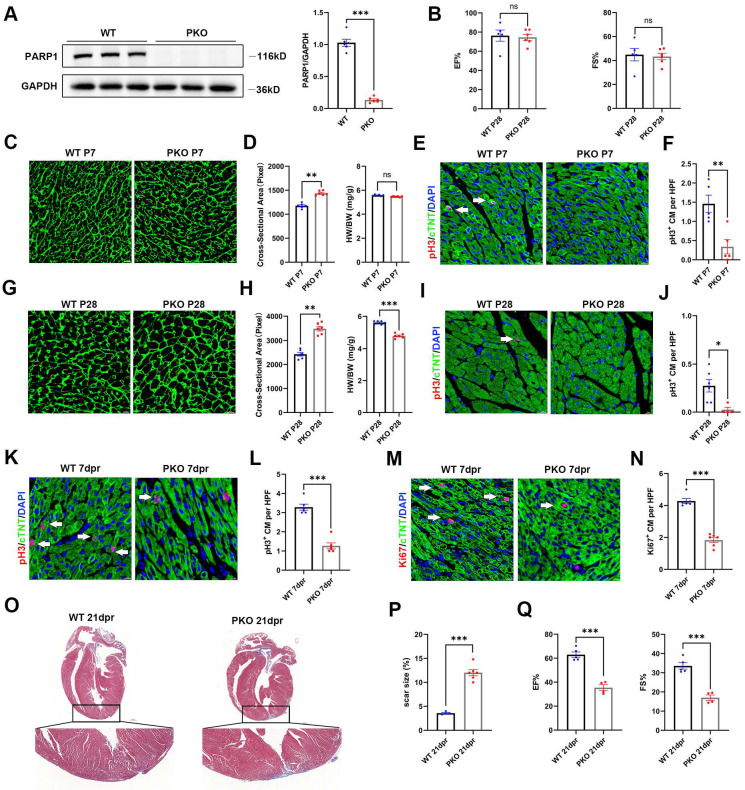
** Knockout of PARP1 inhibits cardiac regeneration in neonatal mice. (A)** Representative western blot images and statistical analysis of PARP1 protein expression in WT mice and PKO mice. GAPDH was used as a loading control (n=6 per group). ***P < 0.001 by unpaired Student's t-test. **(B)** Statistical analysis of ejection fraction (EF) and fractional shortening (FS) of echocardiography in WT mice and PKO mice at P28 (n=5-6 per group). **P < 0.01 by unpaired Student's t-test. **(C and D)** WGA staining and statistical analysis of WGA staining and HW/BW ratio in WT and PKO mice at P7 (n=6 per group). Scale bars, 10μm. **P < 0.01 by unpaired Student's t-test. **(E and F)** Representative immunostaining images and statistical analysis of pH3^+^ (red) cardiomyocyte number in WT and PKO mice at P7 (n=5 per group). Scale bars, 10μm. **P < 0.01 by unpaired Student's t-test. **(G and H)** WGA staining and statistical analysis of WGA staining and HW/BW ratio in WT and PKO mice at P28 (n=6 per group). Scale bars, 10μm. **P < 0.01, ***P < 0.001 by unpaired Student's t-test. **(I and J)** Representative immunostaining images and statistical analysis of pH3^+^ (red) cardiomyocyte number in WT and PKO mice at P28 (n=4-6 per group). Scale bars, 10μm. *P < 0.05 by unpaired Student's t-test. **(K and L)** Representative immunostaining images and statistical analysis of pH3^+^ (red) cardiomyocytes at 7 dpr in WT mice and PKO mice (n=6 per group). Scale bars, 10 μm. ***P < 0.001 by unpaired Student's t-test. **(M and N)** Representative immunostaining images and statistical analysis of Ki67^+^ (red) cardiomyocytes at 7 dpr in WT mice and PKO mice (n=6 per group). Scale bars, 10 μm. ***P < 0.001 by unpaired Student's t-test. **(O and P)** Masson staining images and statistical analysis of heart regeneration and scar size in WT and PKO mice (n=6 per group). Scale bars, 500 μm. ***P < 0.001 by unpaired Student's t-test. **(Q)** Statistical analysis of EF and FS of echocardiography in WT mice and PKO mice at 21 dpr (n=4-5 per group). ***P < 0.001 by unpaired Student's t-test.

To assess the significance of PARP1 in heart regeneration, we employed PARP1-knockout (PKO) mice. By immunoblotting, we validated that PKO mice showed strongly reduced cardiac PARP1 protein abundance relative to WT mice (**Figure 2A**). Cardiac function at P28 was evaluated by echocardiography in PKO and control mice. No significant differences were observed in cardiac function between PKO and control mice (**Figure 2B**). We next measured the heart weight to body weight (HW/BW) ratio and cardiomyocyte size by wheat germ agglutinin (WGA) staining. No obvious differences were observed in the HW/BW ratio between PKO mice and WT mice At P7. However, WGA staining showed a larger cardiomyocyte cross-sectional area of PKO mice than WT mice (**Figure 2C** and** 2D**). PKO mice at P7 had fewer pH3^+^ cardiomyocytes compared to WT (**Figure 2E** and **2F**). Notably, PKO mice exhibited a larger cardiomyocyte size and lower HW/BW ratio than control mice at P28 (**Figure 2G** and** 2H**). Immunostaining showed that PKO mice had fewer pH3^+^ cardiomyocytes than the control mice at P28 (**Figure 2I** and** 2J**), indicating a decrease in cardiomyocyte proliferation and increased cardiomyocyte size in PKO mice.

Next, we examined the regenerative capacities of PARP1 after apical resection (AR) injury by immunofluorescence staining of pH3 and Ki67 with the CM marker anti-cardiac troponin T (cTnT) at 7 days post-resection (dpr). PARP1 ablation significantly suppressed the numbers of pH3^+^ and Ki67^+^ cardiomyocytes (**Figure 2K-2N**). Consistently, Masson's staining analysis displayed a larger scar and fibrosis at the resection site in PKO mice than in their WT littermates (**Figure 2O** and** 2P**). Echocardiography displayed worse heart function with less EF% and FS% in PKO mice than in control mice at 21 dpr (**Figure 2Q**), indicating that PARP1 is necessary for cardiomyocyte proliferation in neonatal heart regeneration.

### PARP1 overexpression promotes heart regeneration in neonatal mice

**Figure 3:**
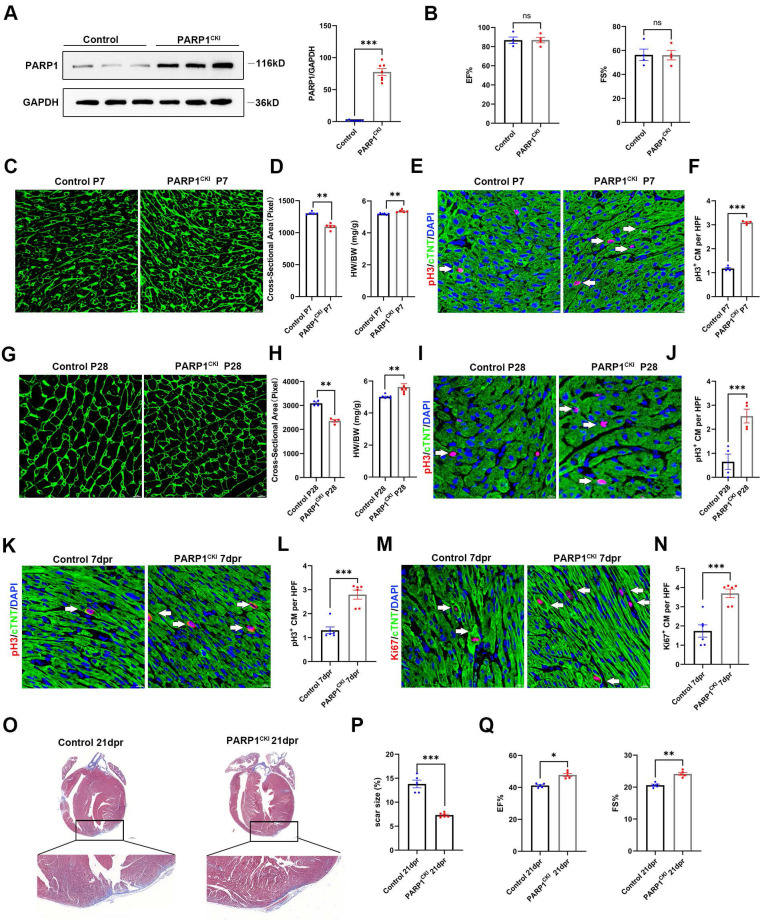
** PARP1 overexpression promotes heart regeneration in neonatal mice. (A)** Representative western blot and statistical analysis of PARP1 protein expression in control mice and PARP1^CKI^ mice. GAPDH was used as a loading control (n=7 per group). ***P < 0.001 by unpaired Student's t-test. **(B)** Statistical analysis of EF and FS of echocardiography at P28 in control mice and PARP1^CKI^ mice (n=4 per group). **(C and D)** WGA staining and statistical analysis of WGA staining and HW/BW ratio in control mice and PARP1^CKI^ mice at P7 (n=5-6 per group), **P < 0.01 by unpaired Student's t-test. **(E and F)** Representative immunostaining images and statistical analysis of pH3^+^ (red) cardiomyocyte number in control mice and PARP1^CKI^ mice at P7 (n=4 per group). Scale bars, 10μm. ***P < 0.001 by unpaired Student's t-test. **(G and H)** WGA staining and statistical analysis of WGA staining and HW/BW ratio in control mice and PARP1^CKI^ mice at P28 (n=4-7 per group). **P < 0.01 by unpaired Student's t-test.** (I and J)** Representative immunostaining images and statistical analysis of pH3^+^ (red) cardiomyocyte number in control mice and PARP1^CKI^ mice at P28 (n=4 per group). Scale bars, 10μm. ***P < 0.001 by unpaired Student's t-test. **(K and L)** Representative immunostaining images and statistical analysis of pH3^+^ cardiomyocytes (red) in control mice and PARP1^CKI^ mice at 7 dpr (n=6 per group). Scale bars, 10 μm. ***P < 0.001 by unpaired Student's t-test. **(M and N)** Representative immunostaining images and statistical analysis of Ki67^+^ cardiomyocytes (red) in control mice and PARP1^CKI^ mice at 7 dpr (n=6 per group). Scale bars, 10 μm. ***P < 0.001 by unpaired Student's t-test. **(O and P)** Masson staining images and statistical analysis of heart regeneration and scar size in control mice and PARP1^CKI^ mice (n=5-6 per group). Scale bars, 500 μm. ***P < 0.001 by unpaired Student's t-test. **(Q)** Statistical analysis of EF and FS of echocardiography in control mice and PARP1^CKI^ mice at 21 dpr (n=5 per group), *P < 0.05, **P < 0.01 by unpaired Student's t-test.

To investigate whether PARP1 promotes heart regeneration, we employed cardiomyocyte-specific knockin of PARP1 (PARP1^CKI^) mice. Western blot analysis confirmed cardiac PARP1 was effectively overexpressed in mice (**Figure 3A**). Echocardiography results at P28 displayed no differences in cardiac function between PARP1^CKI^ mice and control mice (**Figure 3B**). We also measured the HW/BW ratio and found a higher HW/BW ratio in PARP1^CKI^ mice at P7 and P28, and WGA staining revealed a reduction in the cross-sectional areas of cardiomyocyte size in PARP1^CKI^ mice (**Figure 3C** and** 3D, Figure 3G** and** 3H**), as well as increasing number of pH3^+^ cardiomyocytes relative to their littermates by immunostaining (**Figure 3E** and** 3F, Figure 3I** and** 3J**), showing improvements of cardiomyocyte proliferation and reduced cardiomyocyte size in PARP1^CKI^ mice.

Furthermore, PARP1^CKI^ mice and littermates were subjected to AR at P7, and we measured cardiomyocyte proliferation capacity at 7 dpr and cardiac function at 21 dpr. Immunofluorescence staining showed that PARP1 overexpression enhanced cardiomyocyte proliferation at 7 dpr, as indicated by increased numbers of pH3^+^ and Ki67^+^ cardiomyocytes (**Figure 3M-3N**). As shown by Masson staining, PARP1^CKI^ mice had significantly fewer myocardial scars than control mice (**Figure 3O** and** 3P**). Consistently, Echocardiography analysis revealed a noticeable improvement in left ventricular systolic function by showing EF% and FS% increase in PARP1^CKI^ mice relative to their littermates at 21 dpr (**Figure 3Q**). These results suggested that PARP1 increased cardiomyocyte proliferation and neonatal heart regeneration following cardiac injury.

### PARP1 controls cardiomyocyte proliferation in vitro

**Figure 4:**
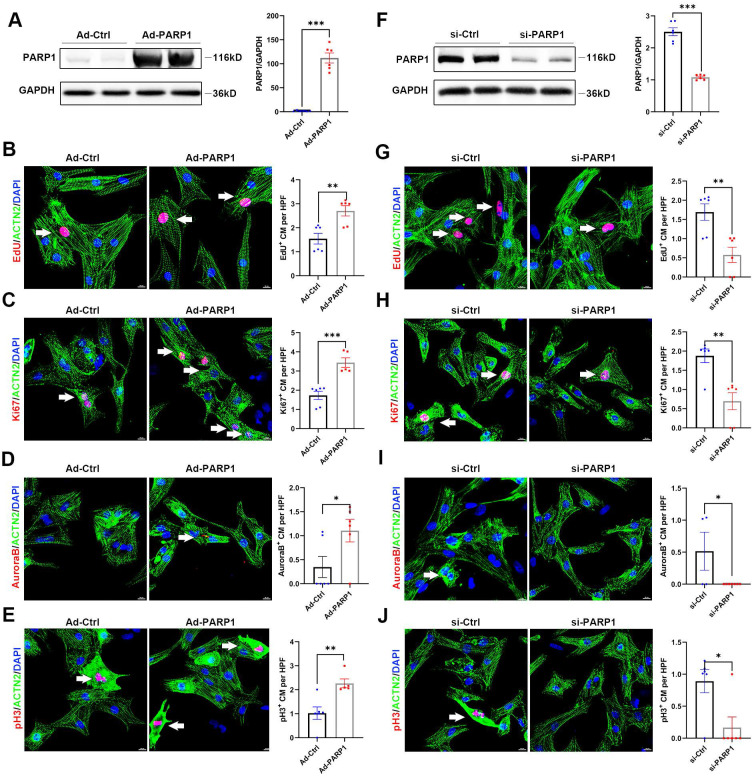
** PARP1 controls cardiomyocyte proliferation in vitro. (A)** Representative western blot images and statistical analysis of PARP1 protein expression in NRCMs infected with Ad-Ctrl or Ad-PARP1. GAPDH was used as a loading control (n=6). ***P < 0.001 by unpaired Student's t-test. **(B-E)** Representative immunostaining images and statistical analysis of EdU^+^, Ki67^+^, Aurora B^+^, and pH3^+^ (red) cardiomyocyte number in Ad-Ctrl or Ad-PARP1 infected NRCMs (n=5-6). Scale bars, 10 μm. *P < 0.05, **P < 0.01, and ***P < 0.001 by unpaired Student's t-test. **(F)** Representative western blot images and statistical analysis of PARP1 protein expression in NRCMs transfected with si-Ctrl or si-PARP1. GAPDH was used as a loading control (n=6). ***P < 0.001 by unpaired Student's t-test. (**G-J**) Representative immunostaining images and statistical analysis of EdU^+^, Ki67^+^, Aurora B^+^, and pH3^+^(red) cardiomyocyte number in si-Ctrl or si-PARP1 transfected NRCMs (n=5-7). Scale bars, 10 μm. *P < 0.05, **P < 0.01, and ***P < 0.001 by unpaired Student's t-test.

Since PARP1 modulation markedly mediated cardiac regeneration in mice, we aimed to analyze whether PARP1 influenced the proliferation of isolated neonatal rat cardiomyocytes (NRCMs) in vitro. First, we infected NRCMs with Ad-PARP1 or Ad-control, and PARP1 overexpression was determined shown in **Figure 4A**, and the transduction efficiency of Ad-PARP1 was verified (**Figure S3**). We stained NRCMs with the cell cycle markers EdU, Ki67, Aurora B kinase, and pH3 to assess the effect of PARP1 on cardiomyocyte proliferation. Immunostaining images displayed that EdU^+^, Ki67^+^, Aurora B^+^, and pH3^+^ NRCMs were significantly increased in Ad-PARP1-infected NRCMs compared with Ad-control NRCMs (**Figure 4B-E**), suggesting that PARP1 promotes cardiomyocyte proliferation in vitro. In contrast, siRNA was employed to silence PARP1 in NRCMs. PARP1 siRNA significantly reduced the PARP1 expression (**Figure 4F**). Notably, the knockdown of PARP1 suppressed the percentages of NRCMs expressing EdU^+^, Ki67^+^, Aurora B^+^, or pH3^+^ markers (**Figure 4G-J**). Furthermore, we used TUNEL assay to examine whether PARP1 affects cardiomyocyte apoptosis, and results showed that the apoptotic level of NRCMs was not influenced by Ad-PARP1 treatment (**Figure S4**). These results suggest that PARP1 exerts a regulatory role in NRCM proliferation.

### PARP1 directly interacts with and poly(ADP-ribosyl)ates HSP90AB1 in cardiomyocytes

**Figure 5:**
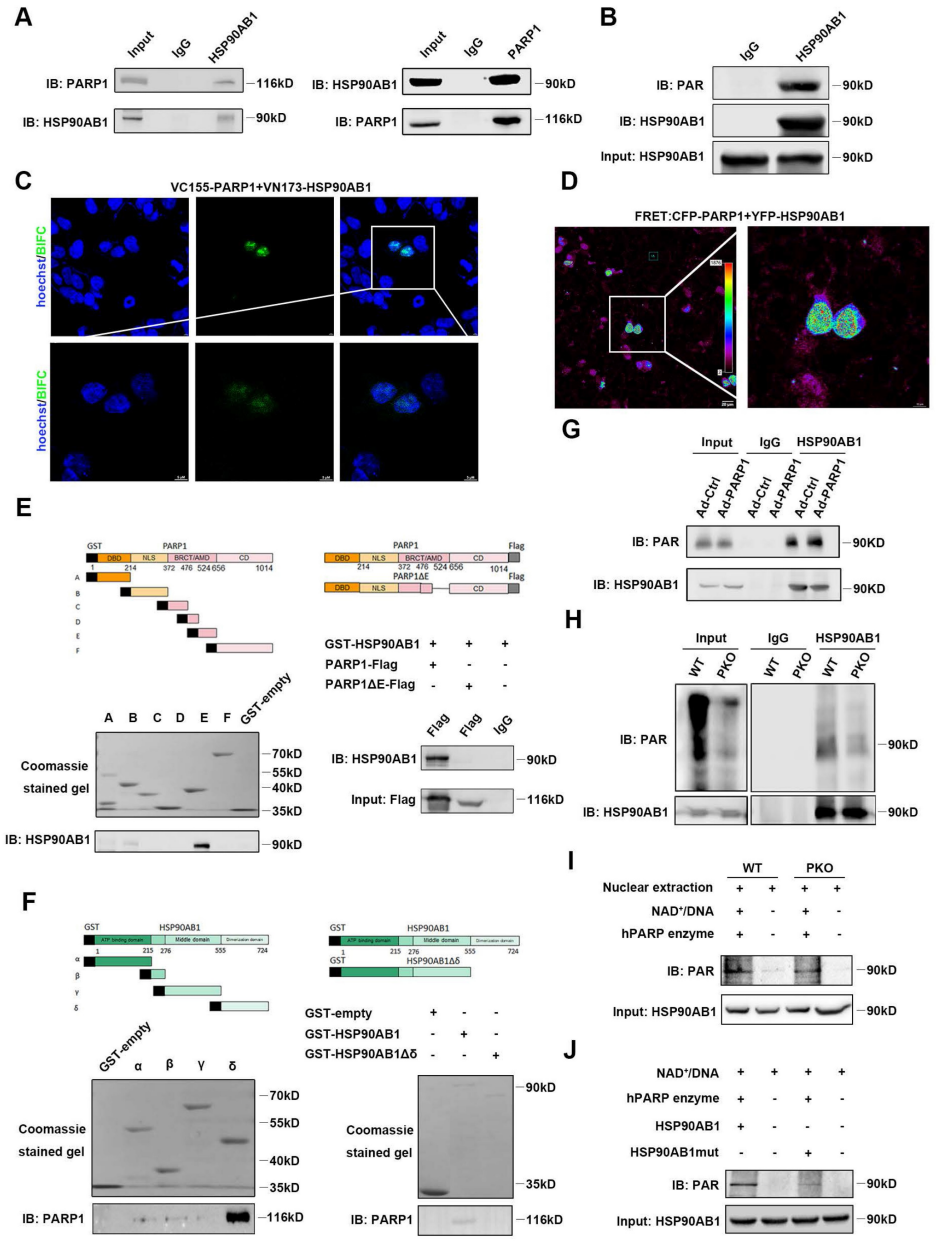
** PARP1 directly interacts with and poly(ADP-ribosyl)ates HSP90AB1 in cardiomyocytes. (A)** Co-IP assay displayed the interaction of PARP1 and HSP90AB1 in NRCMs. **(B)** Co-IP assay showed the poly(ADP-ribosyl)ation of HSP90AB1 in NRCMs. **(C and D)** Representative images of BiFC and FRET assay showing the interaction of PARP1 and HSP90AB1. Scale bars, 10 μm (C) and 20 μm (D). **(E)** GST-pull down assay showed the interaction of PARP1-E domain and PARP1-E domain deletion mutant with HSP90AB1. **(F)** GST-pull down assay showed the interaction of HSP90AB1-δ domain (*Left*) and HSP90AB1-δ domain deletion mutant (*Right*) with PARP1. **(G and H)** Co-IP assay showed the poly(ADP-ribosyl)ation level of HSP90AB1 in NRCMs transinfected with Ad-Ctrl or Ad-PARP1 and in nuclear extracts from WT and PKO mice. **(I)** Co-IP assay showed the poly(ADP-ribosyl)ation level of HSP90AB1 in nuclear extracts from WT or PKO mice heart incubated with active DNA and NAD^+^, respectively. **(J)** Co-IP assay displayed the poly(ADP-ribosyl)ation level of HSP90AB1 in HSP90AB1 and HSP90AB1mut purified protein incubated with active DNA and NAD^+^.

To investigate the underlying mechanism by which PARP1 regulates the proliferation of cardiomyocytes, LC‒MS/MS was performed to identify potential proteins interacting with PARP1. Isolated NCRMs were infected with Ad-PARP1 virus for 48 h, and protein extracts were immunoprecipitated with IgG or PARP1 antibody. Then, the eluted proteins were measured by LC‒MS/MS analysis. HSP90AB1 was top rank in protein-protein interactions with PARP1 (**Table S1**). To confirm this finding, coimmunoprecipitation (Co-IP) assay was conducted in isolated cardiomyocytes and observed that endogenous PARP1 directly interacted with HSP90AB1 or vice versa (**Figure 5A**). BiFC and FRET results demonstrated a strong fluorescence signal, indicating an evident interaction between PARP1 and HSP90AB1 (**Figure 5C** and** 5D**).

Furthermore, we truncated PARP1 into six fragments labeled with GST (A-F) to investigate specific domains interacting with purified HSP90AB1 protein by GST pull-down. We found that the BRCA1 C-terminus/automodification domain of PARP1 strongly bound to HSP90AB1. In contrast, mutation of this fragment could block the PARP1-HSP90AB1 interaction, suggesting that this domain was indispensable for the PARP1-HSP90AB1 interaction (**Figure 5E**). Moreover, we also generated HSP90AB1 truncation into four fragments labeled with GST (α-δ) and tested which fragment interacted with purified PARP1. GST pull-down results revealed that the dimerization domain of HSP90AB1 interacted with PARP1, whereas their interaction diminished by deleting this domain (**Figure 5F**).

A previous study reported that PARP1 activity regulates posttranscriptional modification by poly(ADP-ribosyl)ation. Next, Co-IP assay was conducted to ascertain the potential poly(ADP-ribosyl)ation of HSP90AB1 by PARP1, and results illustrated that HSP90AB1 was poly(ADP-ribosyl)ated in NRCMs (**Figure 5B**). PARP1 overexpression significantly enhanced the poly(ADP-ribosyl)ation level of HSP90AB1 (**Figure 5G**). Moreover, poly(ADP-ribosyl)ated HSP90AB1 was largely abrogated in PARP1-deleted hearts; thus, we suspected that it might be poly(ADP-ribosyl)ated by PARP1 (**Figure 5H**). Indeed, incubation of nuclear extracts from PKO mouse hearts with recombinant PARP1 protein, NAD^+^, and activated DNA led to reduced poly(ADPribosyl)ation of HSP90AB1 relative to WT mice (**Figure 5I**). By LC‒MS/MS analysis, we found and mutated the poly(ADPribosyl)ated site of HSP90AB1 (**Table S2**). Consistently, incubation of purified mutated HSP90AB1 protein with recombinant PARP1 protein, NAD^+^, and activated DNA reduced HSP90AB1 poly(ADP-ribosyl)ation relative to WT HSP90AB1 in a cell-free system (**Figure 5J**). Interestingly, our results displayed poly(ADP-ribosyl)ated HSP90AB1 in PKO mice. Early research also observed a significant level of poly(ADP-ribose) polymerase activity in PARP1-lacking mice, and a short poly(ADP-ribose) polymerase-1 (sPARP1), PARP2 or other enzymes might contribute to the generation of residual ADP-ribose polymers^23^-^25^.

### PARP1 controls cardiomyocyte proliferation through HSP90AB1-CDC37 functional complex formation

**Figure 6:**
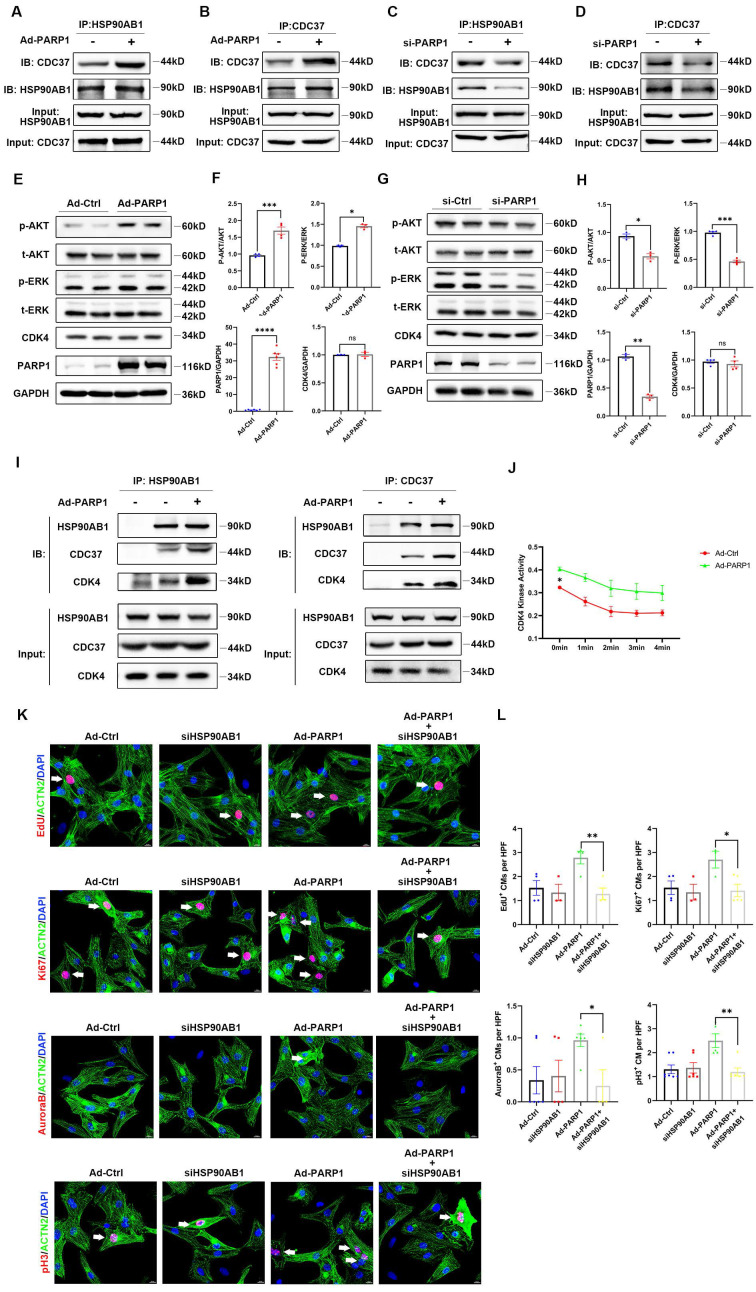
** PARP1 controls cardiomyocyte proliferation through HSP90AB1-CDC37 functional complex formation. (A and B)** Co-IP assay showed the binding between HSP90AB1 and CDC37 in NRCMs infected with or without Ad-PARP1. **(C and D)** Co-IP assay showed the binding between HSP90AB1 and CDC37 in NRCMs infected with or without si-PARP1. **(E and F)** Representative western blot images and statistical analysis of protein expression of p-AKT/AKT, p-ERK /ERK, CDK4, PARP1, and GAPDH in NRCMs infected with Ad-Ctrl and Ad-PARP1 (n=3-6). *P < 0.05, ***P < 0.001, ***P < 0.001 by unpaired Student's t-test. **(G and H)** Representative western blot images and statistical analysis of protein expression of p-AKT/AKT, p-ERK /ERK, CDK4, PARP1, and GAPDH in NRCMs transfected with si-Ctrl and si-PARP1 (n=3-6). *P < 0.05, **P < 0.01, ***P < 0.001 by unpaired Student's t-test. **(I)** Co-IP assay showed the interaction between HSP90AB1, CDC37, and CDK4 in NRCMs infected with or without Ad-PARP1.** (J)** Statistical analysis of CDK4 kinase activity in NRCMs infected with Ad-Ctrl and Ad-PARP1 at different time points (n=3). *P < 0.05 by two-way ANOVA with Bonferroni test.** (K and L)** Representative immunostaining images and statistical analysis of EdU^+^, Ki67^+^, Aurora B^+^, pH3^+^ cardiomyocytes (red) in Ad-Ctrl, si-HSP90AB1, Ad-PARP1, and Ad-PARP1+siHSP90AB1 NRCMs (n=3-7). *P < 0.05, **P < 0.01 by ANOVA followed by the Bonferroni test.

As HSP90AB1-CDC37 complex functions as a molecular chaperone to influence its client protein stability, such as cell cycle kinases, the key regulators related to cell proliferation, we hypothesized that PARP1 might mediate cardiomyocyte proliferation by regulating HSP90AB1-CDC37 chaperone activity. Protein extracts from Ad-PARP1-infected or si-PARP1-treated NRCMs were incubated with IgG, HSP90AB1 antibody, or CDC37 antibody. Co-IP results showed an enhancement of the HSP90AB1-CDC37 interaction by PARP1 overexpression (**Figure 6A** and** 6B**), whereas si-PARP1 repressed HSP90AB1 binding to CDC37 (**Figure 6C** and** 6D**), indicating that PARP1 could modulate the formation of HSP90AB1 and the CDC37 complex. In addition to CDK activity, various signaling pathways were involved in the cell proliferation phenotype, such as the PI3K/AKT pathway and MAPK pathway; thus, we detected changes in these pathways and CDKs by western blotting in isolated NRCMs. The P-AKT/AKT and p-ERK/ERK ratios were significantly increased or decreased, respectively, while the protein level of CDK4 was not altered by PARP1 overexpression or knockdown (**Figure 6E-H**). In addition, AKT inhibitor MK-2206 (HY-10358, MCE, China) or ERK inhibitor PD98059 (HY-12028, MCE, China) partially reduced cardiomyocyte proliferation enhanced by PARP1 overexpression (**Figure S5**). As CDK4 is a crucial client protein of HSP90AB1-CDC37 complex, we measured CDK4 kinase activity with or without PARP1 supplementation. We found an increase in CDK4 binding to HSP90AB1-CDC37 complex and CDK4 kinase activity in the Ad-PARP1 group relative to Ad-Ctrl group (**Figure 6I and 6J**). More importantly, we treated isolated Ad-PARP1-infected NCRMs in the presence or absence of HSP90AB1 siRNA to evaluate cardiomyocyte proliferation by immunofluorescence staining with cell cycle markers.

HSP90AB1 ablation blocked the improvement of cardiomyocyte proliferation by overexpressing PARP1, as indicated by lower percentages of positive cardiomyocyte number by EdU, Ki67, Aurora B, and pH3 immunostaining (**Figure 6K** and** 6L**). Together, these results demonstrated that PARP1 enhanced cardiomyocyte proliferation via the regulation of HSP90AB1.

### PARP1 improves cardiac repair and cardiomyocyte proliferation in adult mice after MI

**Figure 7:**
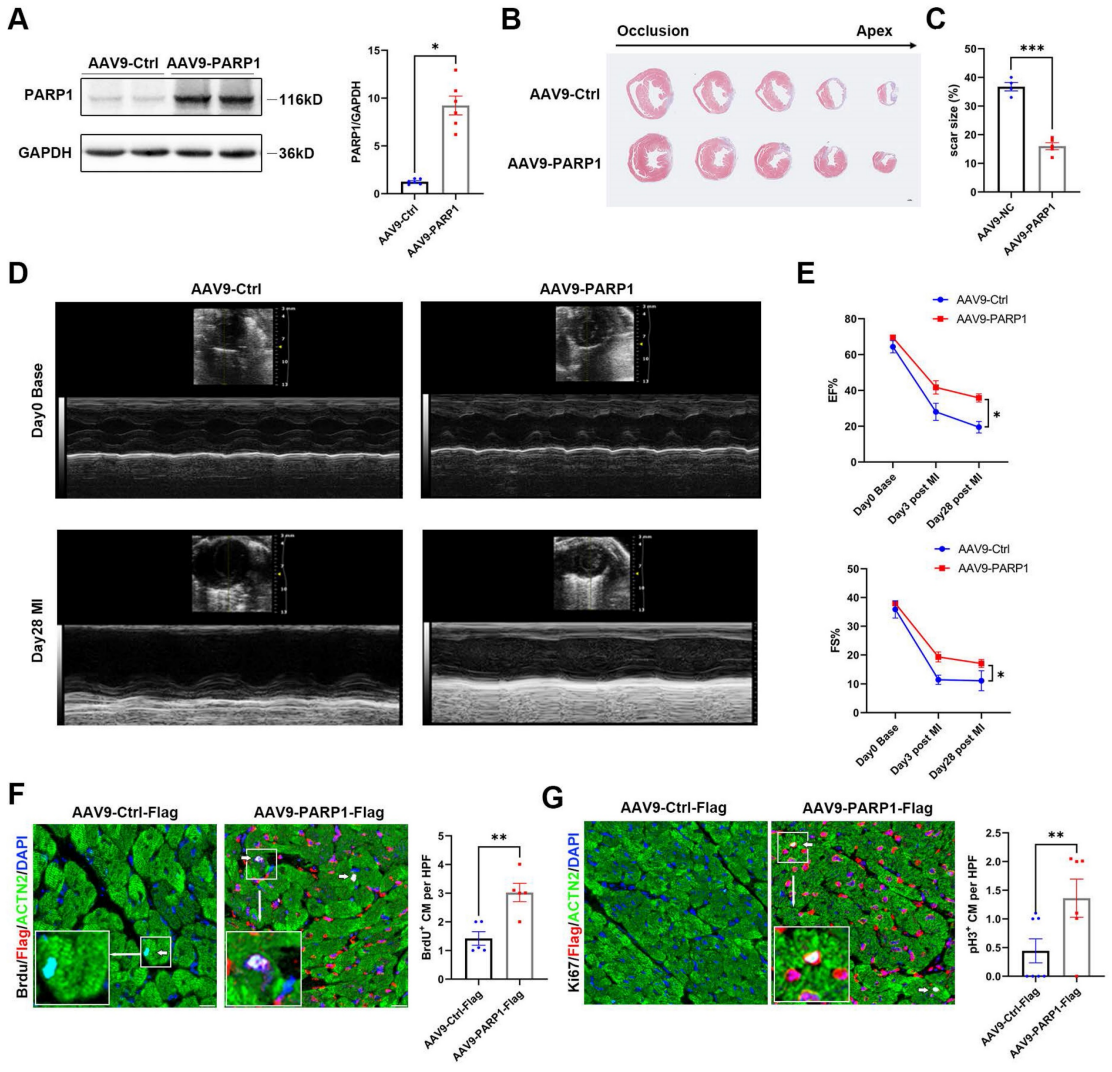
** PARP1 improves cardiac repair and cardiomyocyte proliferation in adult mice after MI.** Representative western blot images and statistical analysis of PARP1 protein expression in C57BL/6J WT mice heart intramyocardially injected with AAV9-Ctrl and AAV9-PARP1. GAPDH was used as a loading control (n=6 per group). *P< 0.05 by unpaired Student's t-test. **(B and C)** Masson staining images and statistical analysis of scar size in AAV9-Ctrl and AAV9-PARP1 mice post MI (n=5-6 per group). Scale bars, 1mm. ***P< 0.001 by unpaired Student's t-test. **(D and E)** Representative images and statistical analysis of EF and FS of echocardiography post-MI and 28 dpi in AAV9-Ctrl and AAV9-PARP1 mice (n=10 per group). **P< 0.01 by unpaired Student's t-test. **(F and G)** Representative immunostaining images and statistical analysis of BrdU^+^ and Ki67^+^ (white) cardiomyocytes in AAV9-Ctrl mice and AAV9-PARP1 mice at 28dpi (n=5-7 per group). Scale bars, 10μm. **P< 0.01 by ANOVA followed by the Bonferroni test.

To test the effect of exogenous PARP1 on cardiac injury in adult animals, we generated myocardial infarction model and intramyocardially injected AAV9-PARP1 into adult WT mice. PARP1 overexpression was verified by western blotting (**Figure 7A**). Masson's staining displayed a reduced scar size in AAV9-PARP1-treated hearts, and echocardiography analysis showed less pronounced systolic dysfunction in AAV9-PARP1 mice at 28 dpi (**Figure 7B-7E, Figure S6**). BrdU and pH3 immunofluorescence at 7 dpi also revealed a higher percentage of positive cardiomyocytes in the AAV9-PARP1 group than in the control group (**Figure 7F** and** 7G**). The findings of this study indicate that the exogenous activation of PARP1 enhances myocardial repair and cardiomyocyte proliferation following MI in adult mice. These findings imply that targeting PARP1 may hold considerable potential as a therapeutic intervention for counteracting cardiac remodeling and preventing heart failure.

## Discussion

Our study revealed that PARP1 has the potential to function as a novel regulator of cardiomyocyte proliferation by interacting with and poly(ADP-ribosyl)ating HSP90AB1. Poly(ADP-ribosyl)ated HSP90AB1 enhanced the interaction with CDC37, which upregulated the kinase activity of cell cycle kinases, thus promoting cardiomyocyte proliferation during cardiac regeneration.

Adult mammalian cardiomyocytes are considered terminally differentiated cells, which are withdrawn from the cell cycle and cease cell proliferation^26^. Therefore, cardiomyocyte re-entry into the cell cycle initiates cardiomyocyte proliferation and heart regeneration. PARP1 has been extensively studied as a target for cancer^27^. Recently, some research has begun to investigate how PARP1 regulates normal cell proliferation and tissue regeneration. An early study revealed that enhancement of PARP1 activity is able to induce the shift from the G0 to G1 phase in quiescent fibroblasts followed by serum stimulation, indicating that PARP1 might be involved in cell cycle regulation 26. Moreover, PARP1 mediates the activity of the E2F1 transcription factor and affects cell cycle progression in embryonic development and tumor growth^27^. A recent study demonstrated that PARP1 is essential for central nervous system development, although poly(ADP-ribosyl)ation inhibits protein‒mRNA interactions, resulting in the upregulation of myelin protein expression and oligodendrocyte proliferation^28^. Our laboratory previously identified that PARP1 plays a fundamental role in liver regeneration and hepatocyte proliferation via Hippo signaling pathway^29^. Interestingly, our previous study provided convincing evidence of a link between PARP1 and heart regeneration. PARP1 could augment the activity of cell division markers and promote cardiomyocyte proliferation after heart injury, providing new insight into the function of PARP1 in heart regeneration.

A particularly striking finding in our study was that the newly identified HSP90AB1 is a poly(ADP-ribosyl)ated target of PARP1 in cardiomyocytes. Poly(ADP-ribosyl)ation mediates cellular and molecular structure and function via multiple regulatory mechanisms, such as inhibition or promotion of protein-protein or nucleic acid interactions, ubiquitylation, and degradation of target proteins, leading to structural conformational changes or transcriptional regulation^18^, ^30^-^34^. We further revealed that poly(ADP-ribosyl)ation of HSP90AB1 enhanced its binding frequency with CDC37, whereas inhibition of poly(ADP-ribosyl)ation blocked the HSP90AB1-CDC37 interaction. Our results reveal that PARP1-related poly(ADP-ribosyl)ation of HSP90AB1 might change its conformational binding site with CDC37, leading to association or disassociation of the protein complex during transcription activity. These findings contribute to further our understanding of the molecular mechanism of poly(ADP-ribosyl)ation in cardiomyocytes.

HSP90AB1 acts as a canonical molecular chaperone that influences protein stability, and together with CDC37, one of HSP90AB1's key cofactors, facilitates the activation of kinase clients such as CDK2, CDK4, and CDK6^35^. Early research has shown that HSP90AB1 regulates liver regeneration, possibly via its chaperone function^36^. He et al. reported that HSP90AB1 controlled skeletal muscle regeneration by regulating MDM2-mediated degradation of P53^37^. We showed that the HSP90 chaperone machinery is also plausibly manipulated in cardiomyocyte proliferation via HSP90AB1-CDC37 and cell cycle kinase interactions. The regulation of the functional HSP90AB1-CDC37 complex involves the participation of various signaling pathways, including TGF-β, MAPK, and PI3K/AKT signaling, where kinases play a critical role in cellular signal transduction^38^. Recent research has shown that the HSP90-CDC37 chaperone complex regulated the autophosphorylation of P38 in cardiomyocytes in the MAPK pathway and mediated cardiomyocyte apoptosis^39^. It is interesting to note that our data revealed that MAPK signaling and PI3K/AKT signaling were activated by PARP1 overexpression, while some cell cycle regulators, such as CDK4, did not present significant changes in protein levels. We speculated that HSP90AB1-CDC37 might interact with CDKs in cardiomyocytes by enhancing kinase activity and cell cycle progression and not altering their protein stability. However, the precise mechanism by which PARP1-dependent poly(ADP-ribosyl)ation mediates the function and interaction of the HSP90AB1-CDC37-client protein remains unclear and needs further investigation.

In our study, we employed immunostaining of significant proliferation markers (pH3, Ki67, EdU, Aurora B) to evaluate cardiomyocyte proliferation in vivo and in vitro, which are commonly used in heart regeneration. However, the detection of proliferation markers varies due to their expression difference in time and space, and monitoring dividing cardiomyocyte is needed in further studies via Mosaic Analysis of Double Markers (MADM) or live-cell imaging platforms. Additionally, our findings demonstrated that activated PARP1 promotes cardiomyocyte proliferation and heart regeneration. However, further investigations are needed to elucidate how PARP1 is regulated in heart regeneration and clarify its upstream pathway.

## Conclusions

In summary, our findings elucidate the significance of PARP1 in heart regeneration, indicating a promising therapeutic target for preventing heart failure after cardiac injury. In addition, this study has identified a novel pathway, HSP90AB1-CDC37, which plays a critical role in cardiomyocyte proliferation, underlying PARP1-induced heart regeneration. The aforementioned findings expand the comprehension of the function of PARP1 and offer novel perspectives on the mechanisms underlying heart regeneration.

## Supplementary Material

Supplementary figures.

Supplementary table 1.

Supplementary table 2.

## Abbreviations

AAV: Adeno-associated virus
ACTN2: Actinin Alpha 2
Ad: Adenovirus
AR: Apical Resection
CDC37: Cell Division Cycle 37
cTNT: Cardiac Troponin T
E2F1: E2F Transcription Factor 1
HSP90AB1: Heat Shock Protein 90 Alpha Family Class B Member 1
LAD: Left Anterior Descending
EF: Ejection Fraction
FS: Fractional Shortening
MAPK: Mitogen-Activated Protein Kinase
MI: Myocardial Infarction
PARP1: Poly(ADP-Ribose) Polymerase 1
pH3: Phosphohistone H3
TGF-β: Transforming Growth Factor Beta
WGA: Wheat Germ Agglutinin
