# Supplementary material for: PARP1 Promotes Heart Regeneration and Cardiomyocyte Proliferation
Source: Int J Biol Sci. 2024 Feb 11;20(5):1602–16. doi: 10.7150/ijbs.85526 (PMC10929182; doi:10.7150/ijbs.85526)
Supplement: Supplementary file 1 — Supplementary figures. [file ijbsv20p1602s1.pdf]

PARP1 Promotes Heart Regeneration and Cardiomyocyte Proliferation

Jiangcheng Shu,[1,2] Shu Yan,[1,2] Chenhui Ju,[1,2] Long Chen,[2] Minglu Liang,[2] Cheng Wang,[2,3]* Kai Huang,[1,2]*

[1]Department of Cardiology, Union Hospital, Tongji Medical College, Huazhong University of Science and Technology, Wuhan, Hubei, China

[2]Clinic Center of Human Genomic Research, Union Hospital, Tongji Medical College, Huazhong University of Science and Technology, Wuhan, Hubei, China

[3]Department of Rheumatology, Union Hospital, Tongji Medical College, Huazhong University of Science and Technology, Wuhan, Hubei, China.

*Corresponding authors:

Cheng Wang, MD, PhD
Department of Rheumatology
Union Hospital, Tongji Medical College
Huazhong University of Science and Technology
1277 Jiefang Ave, Wuhan, Hubei 430022, China
Email: cwangunion@hust.edu.cn

Kai Huang, MD, PhD
Department of Cardiology
Union Hospital, Tongji Medical College
Huazhong University of Science and Technology
1277 Jiefang Ave, Wuhan, Hubei 430022, China
Email: huangkaiunion@gmail.com

Keywords: Cardiomyocyte proliferation, heart regeneration, cell cycle, cell signaling, PARP1

Supplementary Figures

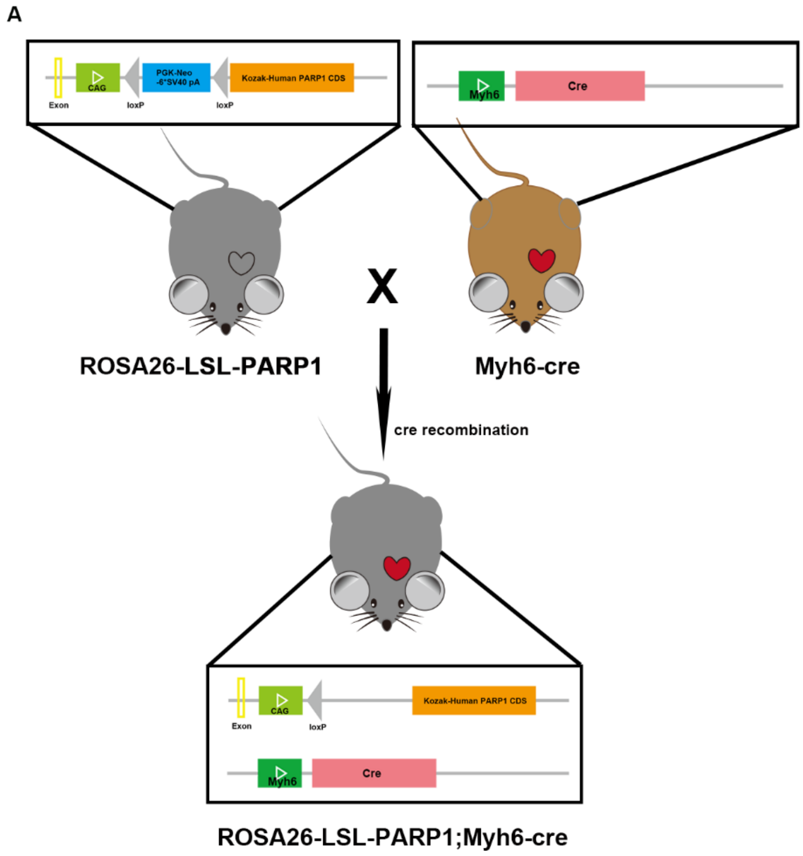

Figure S1 Schematic representation of the conditional knockin of PARP1.

(A) Schematic representation of the conditional knockin of PARP1.

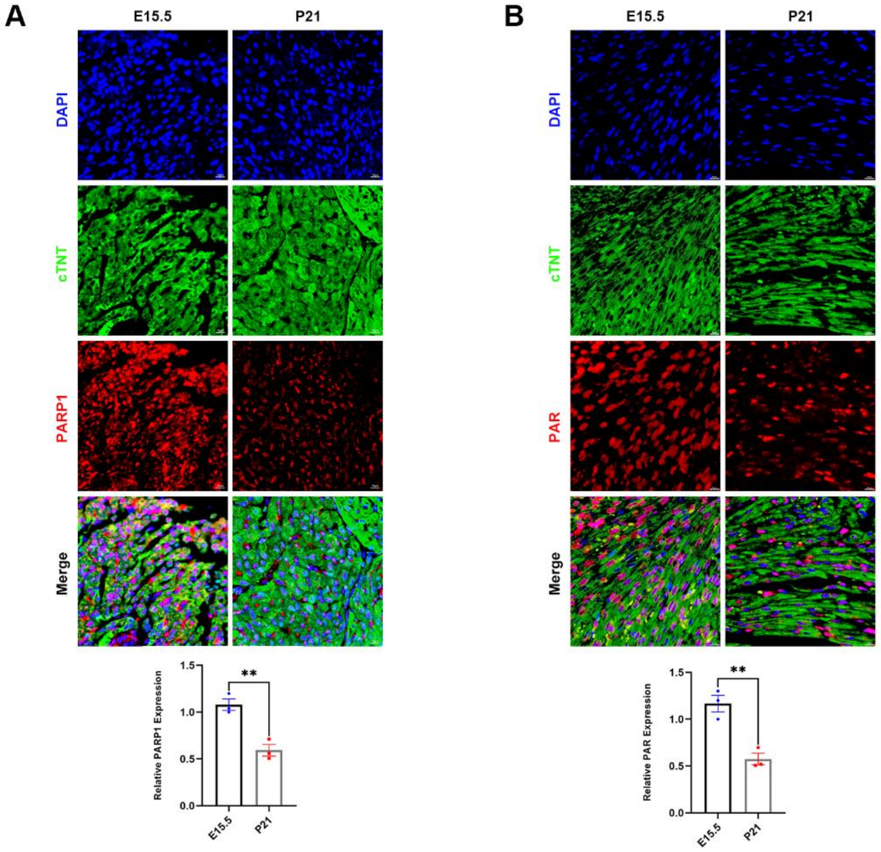

Figure S2 PARP1 and PAR expression decreases in mice heart with age.

(A) Representative immunostaining image and statistical analysis of PARP1 expression level (red) in cardiomyocytes at P21 relative to E15.5 (n=3 per group). Scale bars, 10μm. **P < 0.01 by unpaired Student's test. **(B)** Representative immunostaining image and statistical analysis of PAR expression level (red) in cardiomyocytes at P21 relative to E15.5 (n=3 per group). Scale bars, 10μm. **P < 0.01 by unpaired Student's test.

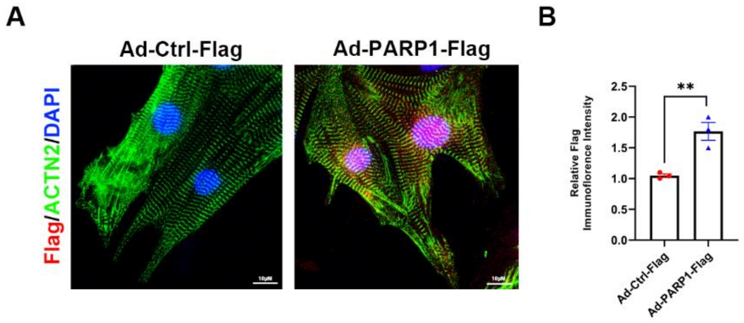

Figure S3 Transduce efficiency of Ad-PARP1-Flag in NRCMs.

(A) Representative images and statistical analysis of transduce efficiency of Ad-ctrl-Flag and Ad-PARP1-Flag infected NRCMs (n=3). **P < 0.01 by unpaired Student's test.

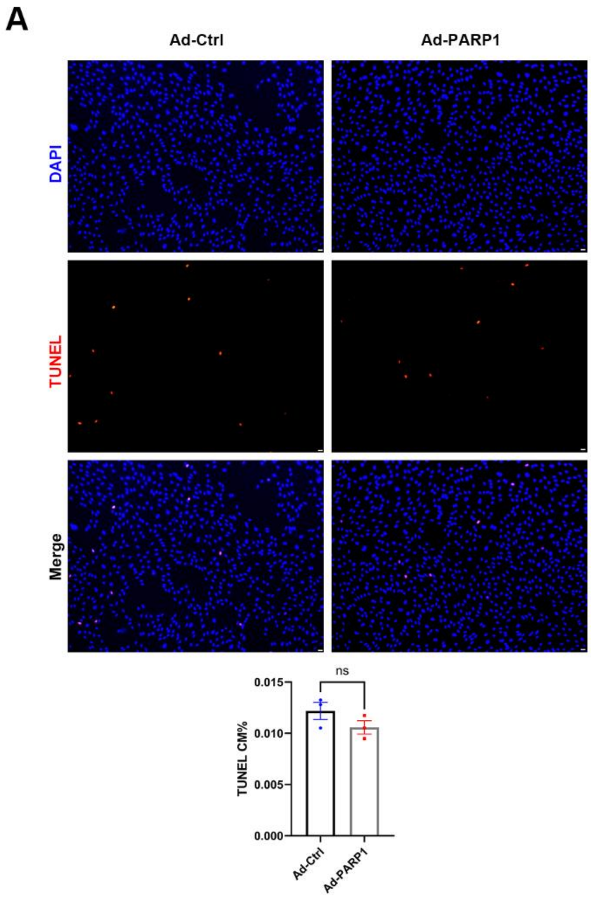

Figure S4 PARP1 overexpression does not affect cardiomyocyte apoptosis.

(A) TUNEL staining and statistical analysis of cell apoptosis between Ad-Ctrl and Ad-PARP1 infected NRCMs (n=3).

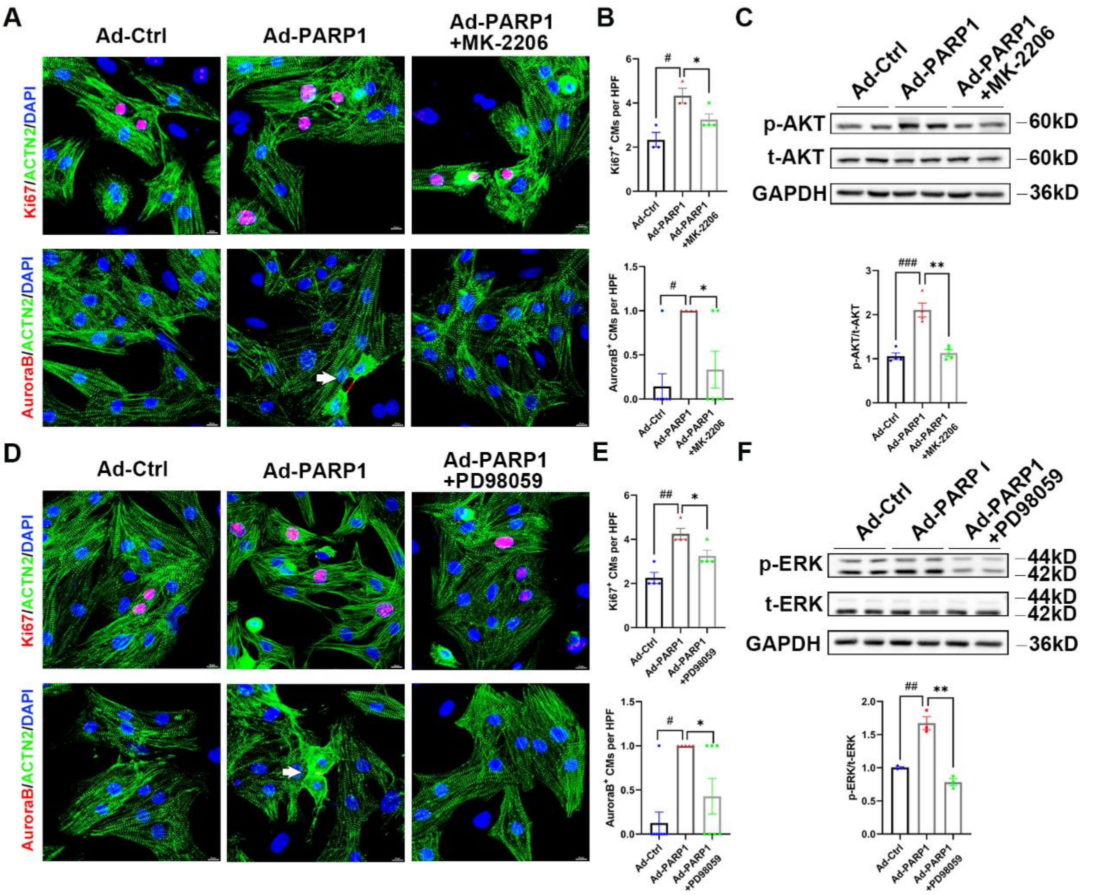

Figure S5 ERK or AKT inhibitor partially hinders the effect of PARP1 on cardiomyocyte proliferation.

(A and B) Representative images and statistical analysis of $Ki67^{+}$, Aurora B^{+} cardiomyocytes (red) in Ad-Ctrl, Ad-PARP1, and Ad-PARP1+MK2206 NRCMs (n=3-6). $^{\#}P < 0.01$ by unpaired Student's test, $^{*}P < 0.01$ by unpaired Student's test. **(C)** Representative western blot images and statistical analysis of p-AKT, t-AKT, and GAPDH protein expression (n=4). $^{\#\#\#}P < 0.001$ by unpaired Student's test, $^{**}P < 0.01$ by unpaired Student's test. **(D and E)** Representative images and statistical analysis of $Ki67^{+}$, Aurora B^{+} cardiomyocytes (red) in Ad-Ctrl, Ad-PARP1, and Ad-PARP1+PD98059 NRCMs (n=3-7). $^{\#}P < 0.01$ by unpaired Student's test, $^{*}P < 0.01$ by unpaired Student's test. **(F)** Representative western blot images and statistical analysis of p-AKT, t-AKT, and GAPDH protein expression (n=3). $^{\#\#}P < 0.01$ by unpaired Student's test, $^{**}P < 0.01$ by unpaired Student's test.

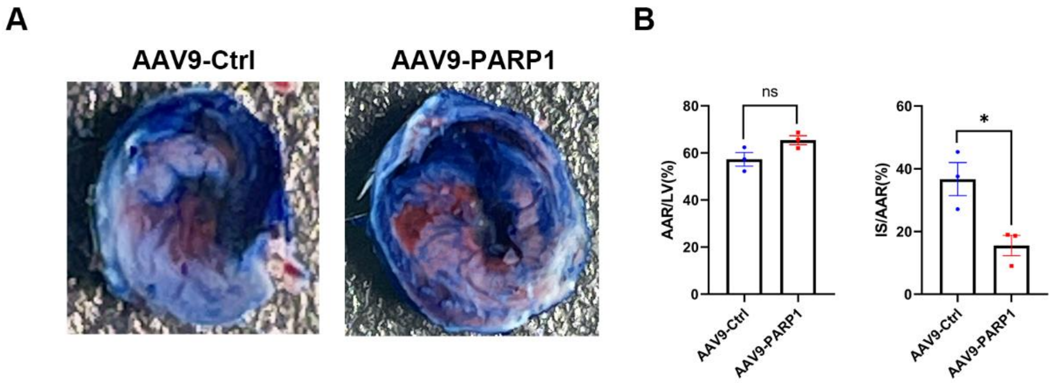

Figure S6 PARP1 improves cardiac repair in adult mice after MI.

(A and B) Representative images of Evans blue-TTC staining of MI and statistical analysis of area at risk (AAR) and infarct size (IS) in AAV9-Ctrl and AAV9-PARP1 group (n=3). *$P < 0.05$ by unpaired Student's test.
